# Age-Related Patterns of Organ-Specific Iron Distribution in Non-Transfusion-Dependent Thalassemia: A Multicenter MRI Study

**DOI:** 10.3390/jcm15187028

**Published:** 2026-09-10

**Authors:** Antonella Meloni, Laura Pistoia, Paolo Ricchi, Filomena Longo, Lorenza Torti, Valerio Cecinati, Elisabetta Corigliano, Michela Zerbini, Luigi Barbuto, Priscilla Fina, Stefania Renne, Vincenzo Positano, Andrea Barison

**Affiliations:** 1 Bioengineering Unit, Fondazione Monasterio, 56124 Pisa, Italy; positano@ftgm.it; 2 Unità Operativa Complessa Ricerca Clinica, Fondazione Monasterio, 56124 Pisa, Italy; laura.pistoia@ftgm.it; 3 Unità Operativa Semplice Dipartimentale Malattie Rare del Globulo Rosso, Azienda Ospedaliera di Rilievo Nazionale “A. Cardarelli”, 80131 Napoli, Italy; paolo.ricchi@aocardarelli.it; 4 Unità Operativa Complessa Talassemie ed Emoglobinopatie, Azienda Ospedaliero-Universitaria Arcispedale S. Anna, 44124 Ferrara, Italy; filomena.longo@ospfe.it; 5 Unità Operativa Semplice Dipartimentale Day Hospital Talassemici, Ospedale “Sant’Eugenio”, 00143 Roma, Italy; lorenza.torti@aslroma2.it; 6 Struttura Semplice di Microcitemia, Ospedale “SS. Annunziata” Azienda Sanitaria Locale Taranto, 74123 Taranto, Italy; valerio.cecinati@asl.taranto.it; 7 Ematologia Microcitemia, Ospedale San Giovanni di Dio—Azienda Sanitaria Provinciale Crotone, 88900 Crotone, Italy; elisabetta.corigliano@asp.crotone.it; 8 Diagnostica per Immagini e Radiologia Interventistica, Ospedale del Delta, 44023 Lagosanto, Italy; m.zerbini@ausl.fe.it; 9 Unità Operativa Complessa Radiologia Generale e di Pronto Soccorso, Azienda Ospedaliera di Rilievo Nazionale “A. Cardarelli”, 80131 Napoli, Italy; luigibarbuto@hotmail.com; 10 Unità Operativa Complessa Diagnostica per Immagini, Ospedale “Sandro Pertini”, 00157 Roma, Italy; priscilla.fina@gmail.com; 11 Struttura Complessa di Cardioradiologia-Unità di Terapia Intensiva Cardiologica, Presidio Ospedaliero “Giovanni Paolo II”, 88046 Lamezia Terme, Italy; stefania.renne@gmail.com; 12 Cardiology and Cardiovascular Medicine, Fondazione Monasterio, 56124 Pisa, Italy; abarison@ftgm.it

**Keywords:** non-transfusion-dependent thalassemia, iron overload, magnetic resonance imaging, age

## Abstract

**Objectives:** This multicenter cross-sectional study investigated the relationship between age and organ-specific iron burden and characterized multi-organ iron phenotypes across different age groups in adults with non-transfusion-dependent thalassemia (NTDT). **Methods:** We retrospectively evaluated 96 adults with NTDT enrolled in the multicenter Italian Extension-Myocardial Iron Overload in Thalassemia network. Hepatic, pancreatic, and cardiac iron burden were assessed by quantitative magnetic resonance imaging (MRI). **Results:** The median age was 41.64 (36.08–53.92) years. Hepatic iron overload (IO) was present in 59.4% of patients and pancreatic IO in 29.2%, whereas no patient exhibited myocardial IO. Age was not associated with liver iron concentration (LIC) or cardiac R2*. A weak positive correlation was observed between age and pancreatic R2* (R = 0.215, *p* = 0.035), although this association was no longer significant after the exclusion of one extreme value. LIC and pancreatic R2* were not correlated. Across age groups (<40, 40–59, and ≥60 years), the prevalence of pancreatic IO increased (22.9%, 28.6%, and 50.0%, respectively), whereas hepatic IO remained stable. Isolated hepatic IO was the most common phenotype overall, while combined hepatic and pancreatic IO was more frequent among patients aged ≥60 years. **Conclusions:** In adults with NTDT, age was not associated with greater hepatic or cardiac iron burden, whereas older patients showed a higher prevalence of pancreatic iron accumulation and combined hepatic–pancreatic iron involvement. These findings highlight the organ-specific heterogeneity of iron distribution in NTDT and support the role of multi-organ MRI assessment beyond LIC evaluation, particularly in older patients.

## 1. Introduction

Thalassemia comprises a heterogeneous group of inherited disorders characterized by reduced or absent synthesis of globin chains, resulting in chronic hemolytic anemia and a wide spectrum of clinical manifestations and disease severity [1,2,3,4]. Within this spectrum, non-transfusion-dependent thalassemia (NTDT) refers to patients who are able to maintain adequate hemoglobin levels without the need for lifelong regular transfusion support [5,6,7]. However, the absence of transfusion dependence does not imply a benign clinical course. NTDT is characterized by a complex pathophysiological interplay between chronic anemia, ineffective erythropoiesis, and progressive iron accumulation [7,8].

The expansion of ineffective erythropoiesis leads to suppression of hepatic hepcidin production, resulting in increased intestinal iron absorption despite the absence of regular transfusion therapy [9,10,11]. Over time, this mechanism promotes progressive iron accumulation in parenchymal tissues [11,12], making iron overload one of the major determinants of long-term morbidity in NTDT and a key target of lifelong disease monitoring.

Historically, the assessment of iron overload in NTDT has focused primarily on liver iron concentration (LIC), as the liver represents the main site of iron storage and LIC is considered the most reliable measure of total body iron burden [13,14]. Indeed, hepatic iron accumulation has been shown to be common in NTDT, although its prevalence may vary according to patient characteristics, disease phenotype, and the use of iron chelation therapy [15,16,17,18]. However, the widespread implementation of quantitative multi-organ magnetic resonance imaging (MRI) has demonstrated that iron deposition is not uniform across tissues. Extrahepatic iron accumulation appears to be less frequent and to follow organ-specific patterns and kinetics that differ from hepatic iron loading. Pancreatic iron overload has been reported in a subset of patients with NTDT [17], whereas myocardial iron accumulation appears to be infrequent [16,18,19,20,21,22].

Advances in clinical management, disease monitoring, and therapeutic strategies have progressively improved survival in patients with NTDT, resulting in an increasing number of adults reaching older ages [5,23]. This changing clinical scenario raises important questions regarding the impact of prolonged disease exposure on tissue iron distribution. Available data regarding the relationship between aging and iron burden remain limited and heterogeneous. While some studies have suggested an association between advancing age and increased hepatic iron burden [15,24], others have not demonstrated a significant relationship between age and liver iron concentration [16,25]. These differences may reflect heterogeneity among cohorts, including variations in age distribution, disease phenotype, severity of iron loading, therapeutic management, and methodological approaches used for LIC assessment. Importantly, previous studies generally included limited numbers of older adults, potentially restricting the assessment of the cumulative effects of prolonged ineffective erythropoiesis and increased intestinal iron absorption over several decades. Moreover, most previous investigations have focused predominantly on hepatic and cardiac iron assessment, while the long-term impact of aging on extrahepatic tissues, particularly the pancreas, remains poorly characterized.

Therefore, it remains unclear whether aging is associated with increased organ-specific iron accumulation in contemporary adult NTDT populations and whether older patients display distinct patterns of multi-organ iron deposition beyond hepatic siderosis.

The aim of this multicenter study was to investigate the association between age and organ-specific iron burden, including hepatic, pancreatic, and cardiac iron accumulation, and to identify distinct multi-organ iron phenotypes across different age groups in adults with NTDT.

## 2. Materials and Methods

### 2.1. Study Population

The Extension-Myocardial Iron Overload in Thalassemia (E-MIOT) project is a nationwide Italian collaborative network involving 66 specialized thalassemia centers and 15 MRI facilities. Participating MRI units apply standardized, validated acquisition and analysis protocols to ensure harmonized assessment of iron overload across sites. Clinical, laboratory, and imaging information from enrolled patients is collected and integrated into a centralized web-based database accessible to participating centers.

For the present retrospective study, we identified 96 consecutive adult patients (age ≥ 18 years) with NTDT. Patients who had transitioned to a regular transfusion regimen (>4 transfusions per year) prior to or at the time of the MRI examination were strictly excluded.

The study was conducted in accordance with the principles of the Declaration of Helsinki. Ethical approval was obtained from the institutional review boards/ethics committees of the participating MRI centers. All patients provided written informed consent for the collection and analysis of clinical and imaging data.

### 2.2. Iron Overload Assessment

MRI exams for iron overload assessment were performed on clinical 1.5T scanners (GE Healthcare, Milwaukee, WI, USA; Philips Healthcare, Best, The Netherlands; Siemens Healthineers, Erlangen, Germany) equipped with phased-array receiver surface coils. All acquisitions were performed during end-expiratory breath-holds with ECG synchronization.

A single mid-axial slice of the liver [26], a minimum of five axial slices covering the entire pancreas [27], and short-axis images of the left ventricle (LV) at basal, mid-ventricular, and apical levels [28] were acquired with multi-echo gradient echo T2* sequences.

T2* images were analyzed by experienced radiologists with more than 10 years of expertise in MRI iron assessment using the validated HIPPO MIOT^®^ software (Version 2.0, Consiglio Nazionale delle Ricerche and Fondazione Monasterio, Pisa, Italy, Year 2015). T2* values were converted into R2* values according to the formula: R2* = 1000/T2*. Hepatic R2* was measured within a standardized circular region of interest (ROI) [26] and converted into liver iron concentration (LIC) using the Wood calibration curve [29]. Hepatic iron overload was defined as an MRI-derived LIC value ≥ 3 mg/g dry weight [14]. Pancreatic R2* assessment was performed by manually placing three ROIs in the head, body, and tail of the pancreas. ROIs were carefully positioned to exclude major vessels, ducts, and areas affected by susceptibility artifacts due to adjacent gastrointestinal gas [27]. The mean of the three regional R2* measurements was considered representative of global pancreatic R2*. A value above 38 Hz was considered indicative of pancreatic iron overload [27]. For myocardial iron evaluation, the LV was divided according to the standardized 16-segment AHA/ACC model [30]. R2* values were calculated for each myocardial segment, and the global cardiac R2* value was obtained by averaging all segmental measurements [28]. A threshold of 31 Hz (equivalent to T2* = 32 ms) was considered the upper limit of normal for cardiac R2* [31]. This cut-off was selected to increase sensitivity for the detection of early myocardial iron deposition [31], rather than using the more conservative threshold of 50 Hz (T2* = 20 ms) [32,33].

### 2.3. Statistical Analysis

Statistical analysis was performed using SPSS version 27.0 (IBM Corp., Armonk, NY, USA).

The normality of continuous variables was assessed using the Kolmogorov–Smirnov test. Continuous variables were expressed as mean ± standard deviation (SD) or median (interquartile range [IQR]), according to their distribution, whereas categorical variables were reported as frequencies and percentages.

Because MRI-derived parameters showed a non-normal distribution, correlations between continuous variables were evaluated using Spearman’s rank correlation coefficient. A multivariable linear regression model was used to examine the adjusted effect of age on pancreatic iron levels, after controlling for potential clinical confounders, including chelation therapy status and splenectomy.

Comparisons between two independent groups were performed using the independent-samples *t*-test or the Mann–Whitney U test as appropriate according to data distribution. For comparisons among multiple independent groups, one-way analysis of variance (ANOVA) or the Kruskal–Wallis test was used, as appropriate. Categorical variables were compared using the χ^2^ test.

A two-sided *p*-value < 0.05 was considered statistically significant.

## 3. Results

### 3.1. Patients’ Characteristics

The main demographic, clinical, and MRI characteristics of the 96 enrolled patients with NTDT are summarized in Table 1.

The study cohort was well balanced by sex, and the participants ranged in age from 19 to 77 years, with a median age of 41.64 (36.08–53.92) years. Fifty-three patients (55.2%) were completely transfusion-naïve, whereas 43 (44.8%) had a history of only sporadic blood transfusions administered during transient clinical conditions, such as pregnancy, surgery, acute infections, or aplastic crises. Thirty-five (36.5%) patients were receiving iron chelation therapy: 16 (45.7%) were treated with desferrioxamine, 2 (5.7%) with deferiprone, and 17 (48.6%) with deferasirox (DFX).

Quantitative multi-organ MRI assessments demonstrated substantial heterogeneity in iron distribution among different organs. The median LIC was 3.72 (2.08–6.27) mg/g dry weight (dw), and hepatic iron overload was detected in 59.4% of the patients. The median global pancreatic R2* value was 33.45 (28.81–40.65) Hz, with pancreatic iron overload identified in 29.2% of the cohort. The median global cardiac R2* was 23.76 (21.89–25.93) Hz, and no patients exhibited evidence of myocardial iron overload.

No correlation was observed between LIC and global pancreatic R2* values (R = −0.001, *p* = 0.992). Serum ferritin was positively correlated with LIC (R = 0.681, *p* < 0.0001), but not with pancreatic R2* (R = −0.048, *p* = 0.659) or cardiac R2* (R = −0.086, *p* = 0.433).

No significant differences were observed between transfusion-naïve patients and those with a history of sporadic transfusions in LIC [3.79 (2.66–6.56) vs. 3.14 (1.67–6.29) mg/g dw, *p* = 0.276], pancreatic R2* [33.05 (29.99–40.79) vs. 34.70 (28.12–39.74) Hz, *p* = 0.906], or cardiac R2* [24.32 (22.01–26.43) vs. 23.26 (21.79–25.44) Hz, *p* = 0.263].

### 3.2. Relationship Between Age and Organ-Specific Iron Parameters

Age was not associated with LIC (R = 0.000, *p* = 0.999) or global cardiac R2* (R = 0.010, *p* = 0.921). In contrast, age showed a weak but statistically significant positive correlation with global pancreatic R2* (R = 0.215, *p* = 0.035). In multivariable linear regression analysis adjusting for chelation status and splenectomy, age remained independently associated with global pancreatic R2* (β = 0.232, *p* = 0.024). Neither chelation therapy (β = 0.069, *p* = 0.530) nor splenectomy (β = 0.036, *p* = 0.747) contributed significantly to the model.

**Figure 1:**
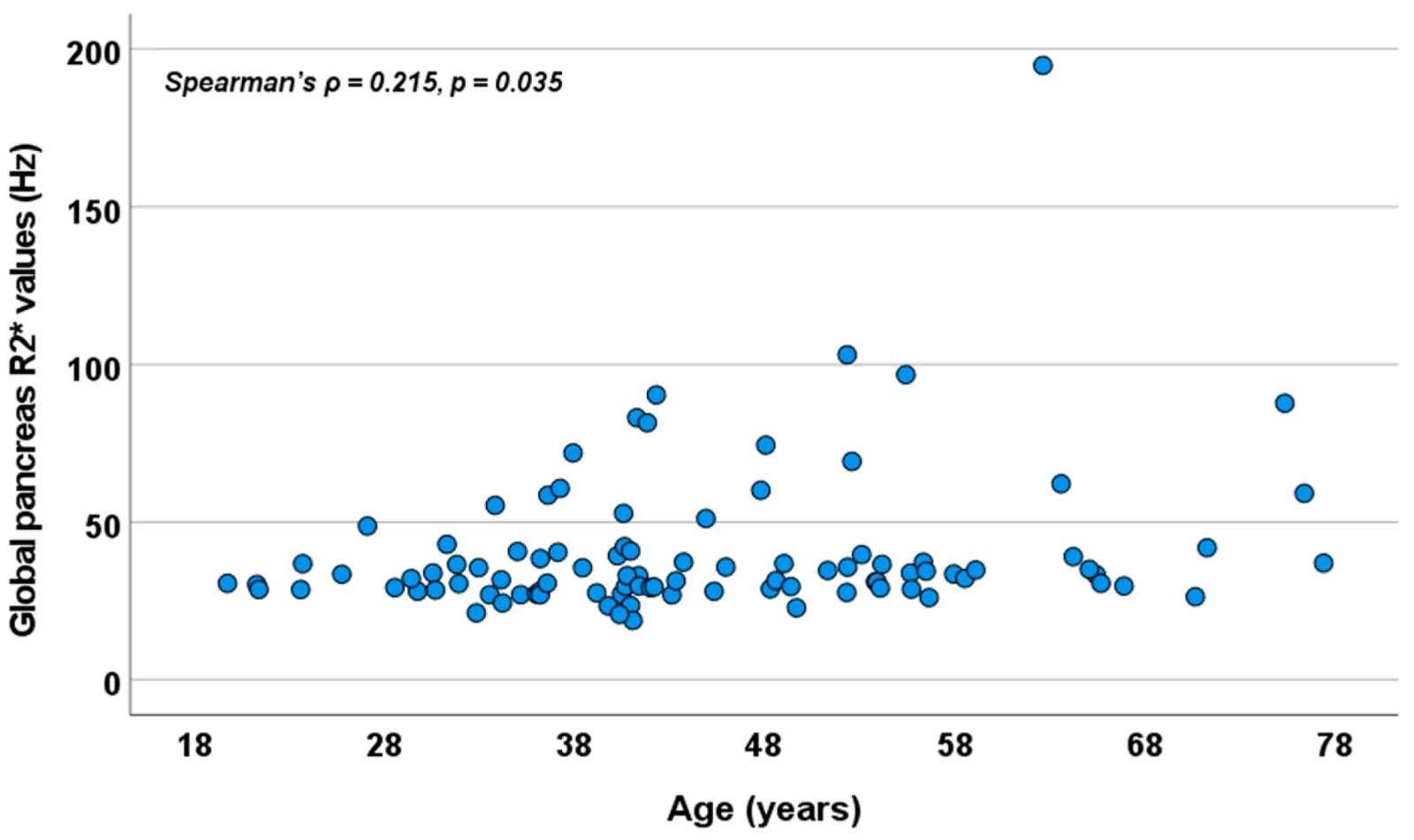
Scatter plot showing the association between age at MRI assessment and global pancreatic R2* values in adult patients with NTDT.

Figure 1 shows the relationship between age at MRI assessment and global pancreatic R2* values. Although pancreatic R2* values displayed considerable inter-individual variability, higher values were observed among older patients. Visual inspection of the scatter plot revealed one patient with an extreme pancreatic R2* value (195 Hz) at the age of 63 years. A sensitivity analysis excluding this observation attenuated the association between age and pancreatic R2*, which no longer reached statistical significance (R = 0.196, *p* = 0.057).

### 3.3. Age-Group Comparison

To further characterize the relationship between age and pancreatic iron accumulation, patients were stratified into three age categories: <40 years (N = 35), 40–59 years (N = 49), and ≥60 years (N = 12). Demographic characteristics, transfusion history, iron chelation therapy, hemoglobin levels, serum ferritin, LIC, and cardiac R2* values were comparable across the three age groups, with no significant between-group differences (Table 2).

Median global pancreatic R2* values showed a progressive increase across age categories, rising from 30.67 (28.12–38.46) Hz in patients younger than 40 years to 33.59 (29.19–40.30) Hz in those aged 40–59 years and 38.06 (31.33–61.43) Hz in patients aged ≥60 years, although the overall difference did not reach statistical significance (*p* = 0.098). A similar trend was observed for the prevalence of pancreatic iron overload, which increased from 22.9% (8/35) in patients younger than 40 years to 28.6% (14/49) in those aged 40–59 years and 50.0% (6/12) among patients aged ≥60 years (*p* = 0.202). In contrast, hepatic iron overload prevalence remained stable across age categories (65.7%, 55.1%, and 58.3%, respectively; *p* = 0.619), and no patient in any age category exhibited myocardial iron overload.

### 3.4. Multi-Organ Iron Phenotypes

To characterize patterns of organ-specific iron distribution, patients were classified into four multi-organ iron phenotypes according to the presence of hepatic and pancreatic iron overload, as no patient exhibited myocardial iron overload. Overall, 28 (29.2%) patients had no evidence of iron overload, 40 (41.7%) had isolated hepatic iron overload, 11 (11.5%) had isolated pancreatic iron overload, and 17 (17.7%) had concurrent hepatic and pancreatic iron overload.

**Figure 2:**
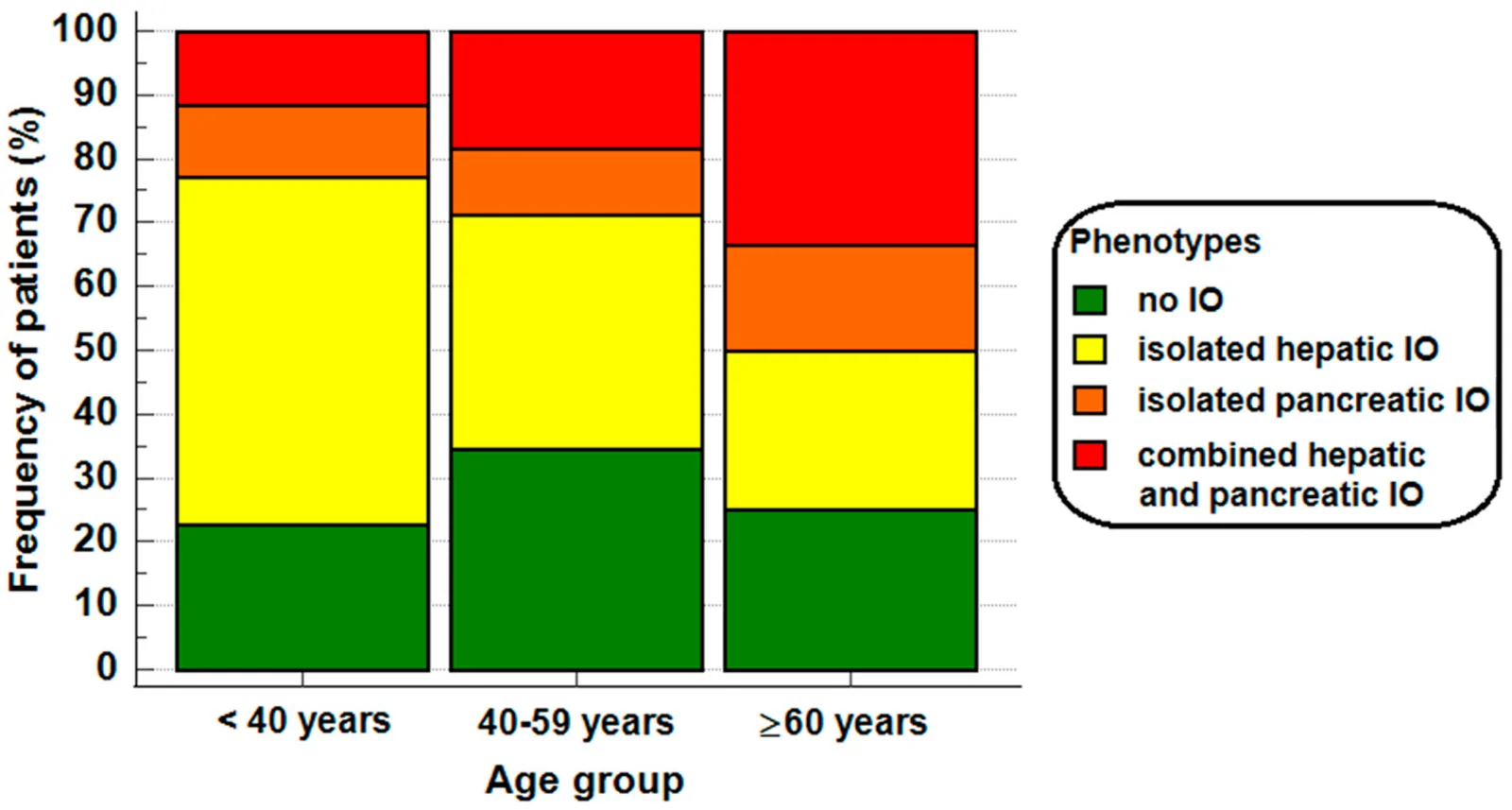
Stacked bar chart showing the distribution of multi-organ (liver and pancreas) iron phenotypes across age categories in adults with NTDT. Patients were classified according to the presence of hepatic and pancreatic iron overload into four phenotypes: no iron overload, isolated hepatic iron overload, isolated pancreatic iron overload, and combined hepatic and pancreatic iron overload. Combined hepatic and pancreatic iron overload was proportionally more frequent among patients aged ≥60 years, although differences among age categories did not reach statistical significance (*p* = 0.392). IO = iron overload.

The distribution of iron phenotypes according to age category is shown in Figure 2. Among patients younger than 40 years, isolated hepatic iron overload was the predominant phenotype (54.3%), whereas concurrent hepatic and pancreatic iron overload was observed in 11.4% of patients. A similar pattern was observed among patients aged 40–59 years, with isolated hepatic iron overload remaining the most frequent phenotype (36.7%), followed by absence of iron overload (34.7%) and concurrent hepatic and pancreatic iron overload (18.4%). Conversely, among patients aged ≥60 years, concurrent hepatic and pancreatic iron overload represented the most frequent phenotype (33.3%), while isolated hepatic iron overload and absence of iron overload were each observed in 25.0% of cases. Isolated pancreatic iron overload accounted for 16.7% of cases.

Although the overall distribution of iron phenotypes did not differ significantly across age groups (*p* = 0.392), combined hepatic and pancreatic iron involvement was more frequently observed among the oldest patients.

## 4. Discussion

In this multicenter study of adults with NTDT, we investigated the relationship between age and organ-specific iron burden using standardized quantitative multi-organ MRI.

In our cohort, iron overload was characterized by predominant hepatic involvement, with hepatic iron accumulation affecting approximately 60% of patients, whereas pancreatic iron accumulation was detected in nearly one-third of the cohort. In contrast, myocardial iron overload was absent across all age groups. This organ involvement profile is consistent with the pathophysiology of NTDT, in which ineffective erythropoiesis leads to reduced hepcidin production, increased intestinal iron absorption, and progressive iron deposition in parenchymal tissues [9,10,11,34]. Importantly, our findings extend previous observations from relatively younger NTDT cohorts [15,16,19,20,22] by showing that this characteristic pattern of organ involvement is maintained in an older population with prolonged disease duration. The persistence of this organ involvement profile despite prolonged disease duration suggests that organ susceptibility in NTDT is primarily influenced by disease-specific mechanisms rather than by cumulative iron exposure alone.

Consistent with the limited available literature [17,35], we did not observe a significant correlation between pancreatic and hepatic iron burden, suggesting that pancreatic iron deposition may not simply reflect overall hepatic iron overload but may follow a partially independent trajectory. Furthermore, the identification of patients with isolated pancreatic iron overload indicates that liver-based assessment alone may underestimate clinically relevant extrahepatic iron involvement and underscores the importance of a comprehensive assessment of organ iron burden in NTDT.

Hepatic iron burden was not associated with age, with comparable LIC values and prevalence of hepatic iron overload across age categories. Consistent with our findings, some studies including predominantly younger NTDT populations have not demonstrated a clear association between age and hepatic iron burden [16,25]. In contrast, other cohorts have reported an association between increasing age and LIC; however, these findings may partly reflect differences in patient characteristics, including the severity of iron overload and the inclusion of transfused patients [15,24]. Our findings extend previous observations by showing that, even in an older NTDT population with prolonged disease duration, age alone does not appear to be a major determinant of hepatic iron accumulation.

An important finding of our study was the observation of a potential relationship between advancing age and pancreatic iron accumulation. Although the association between age and pancreatic R2* was modest and influenced by one extreme observation, pancreatic R2* values showed a progressive numerical increase across age categories, reaching the highest levels in patients aged ≥60 years. This age-related pattern was not observed for hepatic or cardiac iron parameters. These findings suggest that prolonged exposure to increased iron absorption may preferentially affect pancreatic tissue in some patients. However, the substantial variability observed among older individuals indicates that pancreatic iron accumulation is not an inevitable consequence of aging but likely reflects additional biological and disease-related factors. Nevertheless, the higher prevalence of pancreatic involvement observed among older patients may also reflect, at least in part, the long-term persistence of iron accumulated over previous decades. This interpretation is consistent with previous longitudinal observations from the E-MIOT network showing that pancreatic iron overload is more difficult to reverse during chelation therapy than hepatic iron overload [36,37], suggesting that the pancreas may behave as a relatively protected or “sanctuary” compartment for iron accumulation. From a clinical perspective, older patients with NTDT may represent a subgroup requiring particular attention to pancreatic iron assessment and metabolic monitoring, given the reported association between pancreatic iron accumulation and β-cell dysfunction, which may subsequently lead to impaired glucose tolerance and diabetes mellitus [38,39].

The analysis of multi-organ iron phenotypes further emphasizes the complexity of iron distribution in NTDT. Isolated hepatic iron overload was the most common phenotype overall and predominated among younger patients. Conversely, combined hepatic and pancreatic iron overload was proportionally more frequent among patients aged ≥60 years. Although these differences did not reach statistical significance, likely because of the limited number of elderly patients, this distribution suggests that prolonged disease duration may be associated with the emergence of more complex patterns of organ involvement. Prospective longitudinal studies will be required to determine whether these phenotypes represent different stages of iron accumulation over the course of NTDT.

### Limitations

The cross-sectional design does not allow assessment of individual trajectories of iron accumulation over time and limits the ability to disentangle the effects of aging from those of cumulative disease duration, treatment exposure, splenectomy, and prolonged exposure to increased intestinal iron absorption. Although the association between age and pancreatic R2* remained significant after adjustment for chelation status and splenectomy, residual confounding cannot be completely excluded, particularly because detailed information on the duration of and adherence to chelation therapy was not incorporated into the analysis. Additionally, complete genotype data were not available for all participants in the E-MIOT database. As genetic background may influence both the severity and organ-specific distribution of iron loading, residual confounding of the association between age and tissue siderosis cannot be excluded. The limited number of patients aged ≥60 years may have reduced statistical power to detect differences among age categories, particularly for pancreatic iron-related outcomes.

## 5. Conclusions

This multicenter study demonstrates that iron overload in adults with NTDT follows organ-specific patterns, with frequent hepatic involvement, relevant pancreatic accumulation, and no evidence of myocardial iron deposition. Older age was not associated with increased hepatic or cardiac iron burden, whereas older age groups showed a higher frequency of pancreatic involvement and combined hepatic–pancreatic iron phenotypes. These findings support the use of multi-organ MRI beyond LIC assessment alone and suggest that pancreatic evaluation may provide additional information for individualized monitoring strategies, particularly in older adults with NTDT. However, given the cross-sectional design, the potential for residual confounding, and the limited number of participants in the older age groups, these findings should be interpreted with caution. Further prospective longitudinal studies in larger and more comprehensively characterized cohorts, including detailed genotypic information, are warranted to confirm these age-related patterns and clarify their clinical relevance.

## Figures and Tables

**Table 1:** Demographic, clinical, hematological, and MRI characteristics of NTDT patients.

	NTDT Patients (N = 96)
Age (years)	41.64 (36.08–53.92)
Females, N (%)	46 (47.9)
Splenectomy, N (%)	52 (54.2)
Transfusion history, N (%)	
Naïve	53 (55.2)
Past sporadic transfusions	43 (44.8)
Chelated, N (%)	35 (36.5)
Annual mean hemoglobin (g/dL)	9.11 ± 1.07
Annual mean serum ferritin (ng/mL)	400.00 (250.00–538.00)
MRI LIC (mg/g dw)	3.72 (2.08–6.27)
Hepatic IO, N (%)	57 (59.4)
Global pancreas R2* (Hz)	33.45 (28.81–40.65)
Pancreatic IO, N (%)	28 (29.2)
Global heart R2* (Hz)	23.76 (21.89–25.93)
Cardiac IO, N (%)	0 (0.0)

NTDT = non-transfusion dependent thalassemia, N = number, MRI = magnetic resonance imaging, LIC = liver iron concentration, IO = iron overload.

**Table 2:** Demographic and clinical characteristics, laboratory findings, and multi-organ MRI iron parameters stratified by age group.

	Age < 40 yrs (N = 35)	Age 40–59 yrs (N = 49)	Age ≥ 60 yrs (N = 12)	*p*-Value
Females, N (%)	20 (57.1)	21 (42.9)	5 (41.7)	0.390
Splenectomy, N (%)	14 (40)	32 (65.3)	6 (50.0)	0.068
History of sporadic transfusions, N (%)	14 (40)	21 (42.9)	8 (66.7)	0.257
Chelated, N (%)	12 (34.3)	19 (38.8)	4 (33.3)	0.889
Mean hemoglobin (g/dL)	8.92 ± 1.33	9.21 ± 0.87	9.24 ± 0.89	0.402
Mean serum ferritin (ng/mL)	424.00 (242.50–612.50)	380.50 (250.00–509.00)	335.50 (285.00–1048.75)	0.783
MRI LIC (mg/g dw)	4.12 (2.69–6.29)	3.13 (1.69–5.64)	4.25 (2.02–7.72)	0.582
Hepatic IO, N (%)	23 (65.7)	27 (55.1)	7 (58.3)	0.619
Global pancreas R2* (Hz)	30.67 (28.12–38.46)	33.59 (29.19–40.30)	38.06 (31.33–61.43)	0.098
Pancreatic IO, N (%)	8 (22.9)	14 (28.6)	6 (50)	0.202
Global heart R2* (Hz)	24.39 (21.86–26.77)	23.26 (21.98–25.38)	23.24 (21.92–26.06)	0.703
Cardiac IO, N (%)	0 (0.0)	0 (0.0)	0 (0.0)	1.000

N = number, MRI = magnetic resonance imaging, LIC = liver iron concentration, IO = iron overload.

## Data Availability

The data presented in this study are available on request from the corresponding author. The data are not publicly available due to privacy.

## References

[1] Weatherall D.J., Clegg J.B. (2001). The Thalassemia Syndromes.

[2] Angastiniotis M., Lobitz S. (2019). Thalassemias: An Overview. Int. J. Neonatal Screen..

[3] Taher A.T., Weatherall D.J., Cappellini M.D. (2018). Thalassaemia. Lancet.

[4] Sadiq I.Z., Abubakar F.S., Usman H.S., Abdullahi A.D., Ibrahim B., Kastayal B.S., Ibrahim M., Hassan H.A. (2024). Thalassemia: Pathophysiology, Diagnosis, and Advances in Treatment. Thalass. Rep..

[5] Musallam K.M., Rivella S., Vichinsky E., Rachmilewitz E.A. (2013). Non-transfusion-dependent thalassemias. Haematologica.

[6] Shash H. (2022). Non-Transfusion-Dependent Thalassemia: A Panoramic Review. Medicina.

[7] Taher A., Musallam K., Cappellini M.D. (2023). Guidelines for the Management of Non-Transfusion-Dependent β-Thalassaemia.

[8] Saliba A.N., Musallam K.M., Taher A.T. (2023). How I treat non-transfusion-dependent β-thalassemia. Blood.

[9] Tanno T., Miller J.L. (2010). Iron Loading and Overloading due to Ineffective Erythropoiesis. Adv. Hematol..

[10] Ginzburg Y., Rivella S. (2011). beta-thalassemia: A model for elucidating the dynamic regulation of ineffective erythropoiesis and iron metabolism. Blood.

[11] Porter J.B., Cappellini M.D., Kattamis A., Viprakasit V., Musallam K.M., Zhu Z., Taher A.T. (2017). Iron overload across the spectrum of non-transfusion-dependent thalassaemias: Role of erythropoiesis, splenectomy and transfusions. Br. J. Haematol..

[12] Musallam K.M. (2013). Iron overload in non-transfusion-dependent thalassemia. Thalass. Rep..

[13] Anderson G.J., Frazer D.M. (2005). Hepatic iron metabolism. Semin. Liver Dis..

[14] Angelucci E., Brittenham G.M., McLaren C.E., Ripalti M., Baronciani D., Giardini C., Galimberti M., Polchi P., Lucarelli G. (2000). Hepatic iron concentration and total body iron stores in thalassemia major. N. Engl. J. Med..

[15] Huang Y., Yang G., Wang M., Wei X., Pan L., Liu J., Lei Y., Peng, Long L., Lai Y. (2022). Iron overload status in patients with non-transfusion-dependent thalassemia in China. Ther. Adv. Hematol..

[16] Roghi A., Cappellini M.D., Wood J.C., Musallam K.M., Patrizia P., Fasulo M.R., Cesaretti C., Taher A.T. (2010). Absence of cardiac siderosis despite hepatic iron overload in Italian patients with thalassemia intermedia: An MRI T2* study. Ann. Hematol..

[17] Meloni A., Pistoia L., Gamberini M.R., Ricchi P., Cecinati V., Sorrentino F., Cuccia L., Allo M., Righi R., Fina P. (2021). The Link of Pancreatic Iron with Glucose Metabolism and Cardiac Iron in Thalassemia Intermedia: A Large, Multicenter Observational Study. J. Clin. Med..

[18] Meloni A., Pistoia L., Ricchi P., Longo F., Cecinati V., Sorrentino F., Cuccia L., Corigliano E., Rossi V., Righi R. (2024). Multiparametric cardiac magnetic resonance in patients with thalassemia intermedia: New insights from the E-MIOT network. Radiol. Med..

[19] Origa R., Barella S., Argiolas G.M., Bina P., Agus A., Galanello R. (2008). No evidence of cardiac iron in 20 never- or minimally-transfused patients with thalassemia intermedia. Haematologica.

[20] Liguori C., Pitocco F., Di Giampietro I., De Vivo A.E., Schena E., Giurazza F., Sorrentino F., Zobel B.B. (2015). Magnetic resonance comparison of left-right heart volumetric and functional parameters in thalassemia major and thalassemia intermedia patients. Biomed. Res. Int..

[21] Taher A.T., Musallam K.M., Wood J.C., Cappellini M.D. (2010). Magnetic resonance evaluation of hepatic and myocardial iron deposition in transfusion-independent thalassemia intermedia compared to regularly transfused thalassemia major patients. Am. J. Hematol..

[22] Ricchi P., Meloni A., Pistoia L., Spasiano A., Spiga A., Allo M., Gamberini M.R., Lisi R., Campisi S., Peluso A. (2018). The effect of desferrioxamine chelation versus no therapy in patients with non transfusion-dependent thalassaemia: A multicenter prospective comparison from the MIOT network. Ann. Hematol..

[23] Gianesin B., Piel F.B., Musallam K.M., Barella S., Casale M., Cassinerio E., Di Maggio R., Gigante A., Gamberini M.R., Graziadei G. (2025). Prevalence and mortality trends of hemoglobinopathies in Italy: A nationwide study. Haematologica.

[24] Taher A., El Rassi F., Isma’eel H., Koussa S., Inati A., Cappellini M.D. (2008). Correlation of liver iron concentration determined by R2 magnetic resonance imaging with serum ferritin in patients with thalassemia intermedia. Haematologica.

[25] Musallam K.M., Cappellini M.D., Wood J.C., Motta I., Graziadei G., Tamim H., Taher A.T. (2011). Elevated liver iron concentration is a marker of increased morbidity in patients with beta thalassemia intermedia. Haematologica.

[26] Meloni A., Luciani A., Positano V., De Marchi D., Valeri G., Restaino G., Cracolici E., Caruso V., Dell’amico M.C., Favilli B. (2011). Single region of interest versus multislice T2* MRI approach for the quantification of hepatic iron overload. J. Magn. Reson. Imaging.

[27] Restaino G., Meloni A., Positano V., Missere M., Rossi G., Calandriello L., Keilberg P., Mattioni O., Maggio A., Lombardi M. (2011). Regional and global pancreatic T*(2) MRI for iron overload assessment in a large cohort of healthy subjects: Normal values and correlation with age and gender. Magn. Reson. Med..

[28] Meloni A., Restaino G., Borsellino Z., Caruso V., Spasiano A., Zuccarelli A., Valeri G., Toia P., Salvatori C., Positano V. (2014). Different patterns of myocardial iron distribution by whole-heart T2* magnetic resonance as risk markers for heart complications in thalassemia major. Int. J. Cardiol..

[29] Wood J.C., Enriquez C., Ghugre N., Tyzka J.M., Carson S., Nelson M.D., Coates T.D. (2005). MRI R2 and R2* mapping accurately estimates hepatic iron concentration in transfusion-dependent thalassemia and sickle cell disease patients. Blood.

[30] Cerqueira M.D., Weissman N.J., Dilsizian V., Jacobs A.K., Kaul S., Laskey W.K., Pennell D.J., Rumberger J.A., Ryan T., Verani M.S. (2002). Standardized myocardial segmentation and nomenclature for tomographic imaging of the heart: A statement for healthcare professionals from the Cardiac Imaging Committee of the Council on Clinical Cardiology of the American Heart Association. Circulation.

[31] Meloni A., Martini N., Positano V., De Luca A., Pistoia L., Sbragi S., Spasiano A., Casini T., Bitti P.P., Allò M. (2021). Myocardial iron overload by cardiovascular magnetic resonance native segmental T1 mapping: A sensitive approach that correlates with cardiac complications. J. Cardiovasc. Magn. Reson..

[32] Anderson L.J., Holden S., Davis B., Prescott E., Charrier C.C., Bunce N.H., Firmin D.N., Wonke B., Porter J., Walker J.M. (2001). Cardiovascular T2-star (T2*) magnetic resonance for the early diagnosis of myocardial iron overload. Eur. Heart J..

[33] Pennell D.J., Udelson J.E., Arai A.E., Bozkurt B., Cohen A.R., Galanello R., Hoffman T.M., Kiernan M.S., Lerakis S., Piga A. (2013). Cardiovascular function and treatment in beta-thalassemia major: A consensus statement from the American Heart Association. Circulation.

[34] Bou-Fakhredin R., Bazarbachi A.H., Chaya B., Sleiman J., Cappellini M.D., Taher A.T. (2017). Iron Overload and Chelation Therapy in Non-Transfusion Dependent Thalassemia. Int. J. Mol. Sci..

[35] Ning X., Tan S., Peng F., Luo C., Tang C., Xiao F., Peng P. (2024). Organ-Specific Iron Overload in Non-Transfusion-Dependent Thalassemia Patients: Insights from Quantitative MRI Evaluation. Eur. J. Radiol..

[36] Meloni A., Pistoia L., Ricchi P., Allo M., Rosso R., Cuccia L., Casini T., Cecinati V., Serra M., Rossi V. (2023). Prospective changes of pancreatic iron in patients with thalassemia major and association with chelation therapy. Blood Adv..

[37] Ricchi P., Meloni A., Pistoia L., Gamberini M.R., Cuccia L., Allo M., Putti M.C., Spasiano A., Rosso R., Cecinati V. (2024). Longitudinal prospective comparison of pancreatic iron by magnetic resonance in thalassemia patients transfusion-dependent since early childhood treated with combination deferiprone-desferrioxamine vs. deferiprone or deferasirox monotherapy. Blood Transfus..

[38] Noetzli L.J., Mittelman S.D., Watanabe R.M., Coates T.D., Wood J.C. (2012). Pancreatic iron and glucose dysregulation in thalassemia major. Am. J. Hematol..

[39] Kosaryan M., Rahimi M., Darvishi-Khezri H., Gholizadeh N., Akbarzadeh R., Aliasgharian A. (2017). Correlation of Pancreatic Iron Overload Measured by T2*-Weighted Magnetic Resonance Imaging in Diabetic Patients with β-Thalassemia Major. Hemoglobin.

